# Tomato Intake Improves Cognitive Performance and Modulates Functional Brain Networks in Healthy Adults: A Randomized Crossover Clinical Trial

**DOI:** 10.3390/antiox15050644

**Published:** 2026-05-19

**Authors:** Ricardo López-Solís, Carolina Donat-Vargas, Patricia Ramírez-Carrasco, Rocío M. Gutiérrez-Romero, Maria Pérez, Magda Castellví, Beatriz Bosch, Camila Arancibia-Riveros, Alejandro Hinojosa-Moscoso, Carlos Laredo, Emma Muñoz-Moreno, Ana Maria Ruiz-Leon, Rosa Casas, Ramon Estruch, Anna Vallverdú-Queralt, Marina Corrado, Rosa M. Lamuela-Raventós

**Affiliations:** 1 Polyphenol Research Group, Department of Nutrition, Food Science and Gastronomy, Faculty of Pharmacy and Food Sciences, University of Barcelona, 08028 Barcelona, Spain; ricardo.lopez@ub.edu (R.L.-S.); carolina.donat@ub.edu (C.D.-V.); patriciaramirez@ub.edu (P.R.-C.); rogutierrez@ub.edu (R.M.G.-R.); mariaperez@ub.edu (M.P.); camila.arancibia@sjd.es (C.A.-R.); avallverdu@ub.edu (A.V.-Q.); 2 Institut de Recerca en Nutrició i Seguretat Alimentària (INSA-UB), University of Barcelona, 08921 Santa Coloma de Gramenet, Spain; amruiz@recerca.clinic.cat (A.M.R.-L.); rcasas1@recerca.clinic.cat (R.C.); restruch@ub.edu (R.E.); 3 Epidemiology and Public Health Networking Biomedical Research Centre (CIBERESP), Instituto de Salud Carlos III, 28029 Madrid, Spain; 4 Unit of Nutritional Epidemiology, Institute of Environmental Medicine, Karolinska Institutet, 17177 Stockholm, Sweden; 5 Centro de Investigación Biomédica en Red de Fisiopatología de la Obesidad y Nutrición (CIBEROBN), Instituto de Salud Carlos III, 28029 Madrid, Spain; 6 Alzheimer’s Disease and Other Cognitive Disorders Unit, Neurology Service, Hospital Clínic, Institut d’Investigacions Biomèdiques August Pi i Sunyer (IDIBAPS), 08036 Barcelona, Spain; sampol@clinic.cat (M.C.); beabc6@hotmail.com (B.B.); 7 Magnetic Resonance Imaging Core Facility, Institut d’Investigacions Biomèdiques August Pi i Sunyer (IDIBAPS), 08036 Barcelona, Spain; ahinojosa@recerca.clinic.cat (A.H.-M.); laredo@recerca.clinic.cat (C.L.); emunozm@recerca.clinic.cat (E.M.-M.); 8 Department of Internal Medicine, Hospital Clinic, University of Barcelona, Institut d’Investigacions Biomèdiques August Pi i Sunyer (IDIBAPS), 08036 Barcelona, Spain

**Keywords:** tomato, lycopene, antioxidants, carotenoids, cognition, fMRI, BDNF, functional foods

## Abstract

Tomatoes are the major dietary source of lycopene, a carotenoid that crosses the blood–brain barrier and exerts antioxidant and anti-inflammatory effects. However, the impact of tomato consumption on cognitive function in healthy adults remains unclear. This study assessed the effects of concentrated tomato paste on cognitive performance and explored potential mechanisms, including brain-derived neurotrophic factor (BDNF) and functional brain connectivity. A randomized, two-period crossover trial (ClinicalTrials.gov: NCT05891977) was conducted in 47 healthy adults aged 40–55 years assigned to two 3-month interventions separated by a 1-month washout: (a) daily consumption of concentrated tomato paste (0.5 g/kg body weight) and (b) a lycopene-restricted control diet. Cognitive performance was evaluated using validated neuropsychological tests (d2-R, Face-Name Associative Memory Exam, Modified Wisconsin Card Sorting Test), alongside plasma lycopene and BDNF, and resting-state functional magnetic resonance imaging (fMRI). Forty-two participants completed the study. Tomato intake improved selective attention (concentration performance: +7.2 points; processing speed: +8.3 points) and associative memory (face-name matching: +0.8 points). Plasma BDNF showed a borderline increase with tomato intake (mean difference 15.2 ng/mL). Resting-state fMRI revealed changes in brain networks, including reduced connectivity in frontoparietal and auditory networks, contrasting with reductions in the dorsal attention network during the control period. These findings provide evidence that tomato consumption may support cognitive function and modulate brain connectivity in healthy middle-aged adults.

## 1. Introduction

Recent research has highlighted the connection between diet and cognitive function. Dietary patterns rich in antioxidants and anti-inflammatory compounds, such as the Mediterranean diet, have been associated with cognitive benefits and may counteract age-related cognitive decline [1,2,3,4,5]. Tomatoes (*Solanum lycopersicum*), the major dietary source of the carotenoid lycopene, are a central component of this dietary pattern [6]. Among carotenoids, lycopene exhibits the highest singlet oxygen–quenching capacity [7]. It can cross the blood–brain barrier and contribute to reduce oxidative stress and neuroinflammation, while also inhibiting amyloidogenesis, all of which are key processes implicated in neurodegenerative diseases such as Alzheimer’s [6,7,8,9]. In addition, lycopene may contribute to neuroplasticity and brain function by decreasing mitochondrial-oxidative damage and regulating brain-derived neurotrophic factor (BDNF) [6,9,10].

However, evidence for the cognitive effects of tomato intake remains scarce [6]. While some observational studies have reported positive associations between tomato or dietary lycopene intake and cognitive performance [11,12,13], most interventional studies have evaluated tomatoes or lycopene in combination with other foods or bioactive compounds, predominantly in older populations [14,15,16,17,18]. To date, no randomized clinical trial has specifically assessed the effects of a single-food tomato intervention on cognitive outcomes in healthy adults.

To address this gap, we conducted a randomized, crossover clinical trial in healthy adults (aged 40 to 55 years), consisting of two three-month intervention periods: one involving daily consumption of concentrated tomato paste and another following a lycopene-restricted control diet. Cognitive performance was evaluated using validated neuropsychological tests. To explore potential mechanisms linking nutrition and cognition, we measured circulating BDNF as a biomarker of neuroplasticity and assessed changes in brain connectivity using resting-state functional magnetic resonance imaging (fMRI) [3,5]. We hypothesized that regular tomato intake would enhance cognitive performance, increase circulating BDNF concentrations, and modulate functional brain networks, with lycopene as a key bioactive mediator.

## 2. Materials and Methods

### 2.1. Study Design and Population

This randomized, unblinded, two-period crossover trial (MITOS study, ClinicalTrials.gov Identifier: NCT05891977, registered on 17 May 2023) was conducted between November 2023 and December 2024. A sample size of 43 participants was estimated to provide 80% power to detect a 9% relative difference in BDNF concentrations. This calculation was based on an assumed between-individuals standard deviation (SD) of the treatment difference of 0.5, derived from a previous carotenoid intervention [19]. To account for potential dropouts, the planned sample size was increased to 50 participants.

Participants were recruited between September 2023 and April 2024 from Barcelona and surrounding area (~50 km). Eligible participants were healthy adults aged 40–55 years, with no chronic diseases or mental disorders, no alterations in glucose or triglyceride metabolism, body mass index (BMI) < 30 kg/m^2^, non-smokers, and no indication of problematic alcohol use (Alcohol Use Disorders Identification Test [AUDIT] score < 8). Eligibility criteria were verified through a self-reported screening questionnaire and a medical history interview during the inclusion visit. Global cognitive function was assessed using the Montreal Cognitive Assessment (MoCA), with a cutoff score > 26.

Participants were randomly assigned to one of two sequences (AB or BA). The random allocation sequence was generated using an online platform, with a single block of 50 random allocations (AB or BA), and participants were assigned sequentially according to enrollment order. Due to the nature of the intervention, allocation was not concealed, and both participants and study personnel were aware of the assigned condition. Outcome assessors for biological sample analyses (including plasma lycopene and BDNF quantification) and fMRI data were blinded to the intervention assignment.

### 2.2. Settings and Location

Study activities were conducted in different facilities of the Hospital Clínic de Barcelona and its associated research institute, IDIBAPS (Institut d’Investigacions Biomèdiques August Pi i Sunyer), in Barcelona, Spain. Fasting blood samples were collected at the hospital’s outpatient clinic. Samples were immediately processed and either disposed or stored for further analyses according to the experimental protocol. Immediately after, participants moved to the hospital research unit to complete the remaining assessments, including cognitive testing, dietary questionnaires, and body composition analysis. Each study visit lasted approximately 1.5 h. fMRI scans were scheduled separately and conducted at the IDIBAPS Magnetic Resonance Imaging Core Facility. All outcomes and potential confounders were assessed at four time points, corresponding to the beginning and end of each intervention period.

### 2.3. Interventions

Participants completed two dietary interventions, each lasting three months, separated by a one-month washout period. One week before the start of the study, participants were asked to avoid lycopene-rich foods (tomato, watermelon, papaya, grapefruit, guava, and any products containing these ingredients). During the washout, participants followed their habitual diet for three weeks, and during the final week they were asked to follow the same lycopene restriction applied before the first intervention. This schedule was based on pharmacokinetic evidence showing that lycopene has a plasma half-life of approximately 5.3 days for the *trans*-isomer and 8.8 days for the *cis*-isomers [20]. Given these elimination rates, a four-week washout was deemed sufficient for plasma lycopene concentrations to return close to baseline levels.

The interventions were as follows: (A) Tomato intervention: habitual diet plus concentrated tomato paste (0.5 g/kg body weight daily). The paste (supplied by the research team) was consumed raw or cooked with regular meals. (B) Control intervention: a low-lycopene diet in which participants limited tomatoes and other lycopene-rich foods. Participants were instructed not to modify their habitual diet, physical activity, or lifestyle during the study and received written guidance and individual recommendations to minimize lycopene intake, including a list of foods to avoid, suggested food replacements (e.g., substituting watermelon with melon, given their shared seasonality), and a recipe booklet providing paired meal options: preparations using tomato paste for the tomato intervention and equivalent alternatives without tomato for the control period. Plasma lycopene concentrations were measured as a reliable biomarker of adherence to both interventions (tomato and control). No formal harms assessment was prespecified or systematically conducted during the study. An overview of the study design is provided in Supplementary Data, Figure S1.

### 2.4. Tomato Paste Composition

The tomato paste was commercially produced by the CONESA Group (Conservas Vegetales de Extremadura, S.A., Villafranco del Guadiana, Spain) and contained only tomato and salt. Physicochemical characteristics and nutritional composition data were provided by the manufacturer.

#### Carotenoid Profile of Tomato Paste

Tomato paste was freeze-dried and ground into a powder prior to analysis. Carotenoids were extracted as described by Rinaldi et al. [21] and Vallverdú-Queralt et al. [22], with minor modifications. Briefly, 3 g of freeze-dried powder was resuspended in 30 mL of ethanol: *n*-hexane (Panreac AppliChem, Castellar del Vallès, Spain) extraction solution (4:3, *v*/*v*). The suspension was vortexed for 1–2 min, sonicated for 10 min on ice, and centrifuged for 20 min at 2480× *g* at 4 °C. The apolar phase was transferred to an amber flask, and the extraction was repeated twice. The supernatants were combined and evaporated to dryness under nitrogen flow (Techne Sample Concentrator, Stone, Staffordshire, UK). The residue was reconstituted with 1 mL of methyl tert-butyl ether (MTBE; Panreac AppliChem, Castellar del Vallès, Spain), filtered through a 25 mm, 0.22 μm polytetrafluoroethylene (PTFE) filter (Teknokroma, Sant Cugat del Vallés, Spain) into an amber vial, and stored at −80 °C until high-performance liquid chromatography (HPLC) analysis.

Carotenoids were quantified using an ACQUITY ultra-performance liquid chromatography (UPLC) system coupled to a diode array detector (DAD; Waters Corporation^®^, Milford, MA, USA) [22]. The method was previously validated by confirming identification using liquid chromatography–tandem mass spectrometry (LC-MS/MS) [21]. Separation was carried out on a C30 column (250 × 4.6 mm, 5 μm; YMC™, Waters Co., Milford, MA, USA) at 25 °C, with a flow rate of 0.6 mL/min. The mobile phase consisted of methanol (A) and MTBE: methanol (8:2 *v*/*v*) (B). The gradient used to separate the analytes was as follows: 0 min, 90% A; 10 min, 75% A; 20 min, 50% A; 25 min, 30% A; 35 min, 10% A; 43 min, 6% A; 48 min, 6% A; 50 min, 90% A; 57 min, 90% A. The injection volume was 10 μL. The DAD detector was used in the range of 295 to 600 nm, and the chromatograms were acquired at a wavelength of 295, 350 and 450 nm.

Quantification was performed by UPLC-DAD using external calibration curves (at least five concentration levels) for α-carotene, β-carotene, lycopene, and phytoene. *Z*-isomers were quantified using the calibration curve corresponding to the all-*E*-isomers. Results were expressed as mg/100 g of tomato paste (mean ± standard error [SE], *n* = 3). Total carotenoids, lycopene, *Z*-lycopene, and β-carotene were calculated as the sum of the corresponding individual compounds.

### 2.5. Plasma Lycopene Concentrations

Plasma lycopene was quantified using an ACQUITY UPLC system (Waters Corporation^®^, Milford, MA, USA) coupled to a DAD detector. Fasting blood samples were collected in ethylenediaminetetraacetic acid (EDTA) tubes (BD Vacutainer®, Franklin Lakes, NJ, USA) and centrifuged at 1.500× *g* for 15 min at 4 °C to separate the plasma, which was stored at −80 °C until analysis.

Lycopene was extracted from plasma by the liquid–liquid extraction method described by Colmán-Martínez et al. [23] with minor modifications. Briefly, 500 μL of thawed plasma was mixed with 500 μL of ethanol and 1 mL of *n*-hexane containing butylated hydroxytoluene (BHT, 100 mg/L; Sigma-Aldrich, St. Louis, MO, USA). β-apo-8’-carotenal (Sigma-Aldrich, St. Louis, MO, USA) was used as an internal standard at a final concentration of 10 μg/mL. Samples were vortexed for 1 min and centrifuged at 2070× *g* for 5 min at 4 °C. The upper organic phase was transferred to a new tube, and the aqueous phase was re-extracted with an additional 1 mL of *n*-hexane/BHT. The organic phases were pooled and evaporated to dryness under a gentle stream of nitrogen at room temperature. The samples were reconstituted in 125 μL of methanol:MTBE (1:1 *v*/*v*) in amber glass vials, and chromatographic analysis was performed immediately. A calibration curve was prepared using human plasma as a blank, β-apo-8’-carotenal (10 µg/mL) as an internal standard, and a lycopene standard (Extrasynthese, Genay, France).

The chromatographic separation of lycopene was performed as described by Vallverdú-Queralt et al. [24] using a C30 reverse-phase column (250 × 4.6 mm, 5 µm, YMC™, Waters Co., Milford, MA, USA) maintained at 25 °C. The mobile phase consisted of A) water/MTBE/methanol (4:26:70, *v*/*v*/*v*) and B) water/MTBE/methanol (4:90:6, *v*/*v*/*v*). The gradient program used for solvent B was (t (min), %B): (0.0, 26); (23.0, 90); (25.0, 90); (27.0, 26); (32.0, 26). The injection volume was 15 µL and flow rate was 1 mL/min. Plasma lycopene was quantified against a standard lycopene calibration curve (0.1–25 µg/mL; R2 = 0.997). Values were expressed as µmol/L using the molecular weight of lycopene (536.9 g/mol).

### 2.6. Plasma BDNF Concentrations

Plasma BDNF concentrations were quantified using a human BDNF sandwich enzyme-linked immunosorbent assay (ELISA) kit (Thermo Fisher Scientific, Waltham, MA, USA; cat. no. EH42RB). Samples were diluted 1:25, and the analysis was performed according to the manufacturer’s instructions. Absorbance was measured at 450 nm, and concentrations were determined by interpolation on a BDNF standard curve (0–16 ng/mL) fitted to a four-parameter logistic model. Duplicate measurements were averaged for each sample.

### 2.7. Cognitive Assessment

The d2-R test was used to measure selective attention, concentration, visual processing speed, inhibition, and impulsiveness. In this paper-and-pencil task, rows of the letters “d” and “p” were presented, each with one to four small dashes above or below. Participants were instructed to quickly mark only the “d” letters with two dashes, completing 14 lines within 20 s per line [25]. Performance was assessed based on the number of correct targets, errors, and processing speed.

The Modified Wisconsin Card Sorting Test (M-WCST) assessed executive functions, particularly cognitive flexibility, strategy shifting, and the ability to modulate impulsive responses. Participants matched response cards to one of four reference cards based on color, shape, or number. The correct matching rule was not disclosed and had to be inferred from feedback (correct/incorrect). After six consecutive correct responses, the sorting rule changed, requiring participants to adapt their strategy and identify the new criterion. The test ended when six categories were completed (i.e., six sets of six consecutive correct responses) or when all 48 response cards had been used. The M-WCST is a modified version of the traditional Wisconsin Card Sorting Test that eliminates ambiguity by excluding response cards sharing more than one attribute with the stimulus card [26]. Performance was evaluated by the number of categories completed, total errors, perseverative errors (continued use of a previously incorrect rule despite negative feedback), and the Executive Function Index, calculated through the TEAcorrige online scoring system [27].

The Face-Name Associative Memory Exam (FNAME) assessed associative memory involving both verbal and visual domains. Participants learned 12 face-name pairs, each displayed for 5 s, while judging whether the name “fit” the face to enhance attention. After a 15 min delay, memory was tested through three tasks: face recognition (identifying the learned face among two distractors), first letter recall of the associated name, and face-name matching (choosing the correct name from three options). Each task was scored out of 12, and the total associative memory score was the sum of the three subscores [28]. A different version of the test was used at each assessment to minimize learning effects.

### 2.8. Neuroimaging Analysis

A subsample of 14 participants (9 women, 5 men) underwent neuroimaging on a 3-tesla (3T) Siemens PRISMA scanner (Erlangen, Germany). This subsample was determined by participants’ availability, as participation required additional informed consent and separate scheduling, with some participants declining due to anxiety related to magnetic resonance imaging procedures or the additional time commitment involved. Acquisitions included T1-weighted structural imaging (repetition time [TR] = 2.5 s, echo time [TE] = 4.37 ms, voxel size: 1 × 1 × 1 mm) and resting state fMRI (TR = 800 ms, TE = 37 ms, total volumes = 450; voxel size: = 2 × 2 × 2 mm). Resting state fMRI data were pre-processed including: (1) slice-timing correction with FMRIB Software Library (FSL, version 6.0.7.1) [29]; (2) estimation of motion parameters using Statistical Parametric Mapping (SPM, version SPM12) [30], together with framewise displacement (FD) and the temporal derivative of time courses (DVARS) computation with FSL [31]; (3) detection of motion outliers with quality criteria of mean FD > 0.5 mm or more than 10% of volumes with FD > 0.5 mm; (4) elastic registration to the T1-weighted image to correct for echo-planar imaging (EPI) distortion using Advanced Normalization Tools (ANTs, version 2.4.4) [32]; (5) elastic registration into the standard Montreal Neurological Institute (MNI) space using ANTs; (6) spatial smoothing for denoising (sigma = 3 mm), band-pass filtering (0.01–0.15 Hz) and motion-related regressor removal using tools from the nilearn library (version 0.10.1). No images were excluded from the analysis due to motion. Visual inspection of the raw and processed images was performed during the pipeline to ensure image quality.

### 2.9. Demographic, Health, and Lifestyle Data

At enrollment, participants completed a general health questionnaire administered by the research team to collect sociodemographic information (e.g., marital status, education), medical history, medication and supplement use, and lifestyle habits. All other assessments were conducted at the four evaluation time points described in Section 2.2. Dietary intake was assessed using a validated food frequency questionnaire (FFQ) [33]. Physical activity and sleep were objectively measured with accelerometry (ActiGraph wGT3X-BT^®^, Pensacola, FL, USA). Participants wore the device on their non-dominant wrist for seven consecutive days prior to each evaluation visit, including during sleep. Data were processed using ActiLife software (version 6.13.4, ActiGraph LLC, Pensacola, FL, USA), applying a cut-off of ≥2690 counts/min to classify moderate-to-vigorous physical activity [34]. Body composition was assessed using bioelectrical impedance analysis (Omron BF511, Kyoto, Japan). Blood pressure was measured twice in both arms (with a third measurement if discrepant) using an automated sphygmomanometer (Omron M6, Kyoto, Japan). Reported values reflect the mean of the available measurements. Fasting plasma biochemical markers (glucose, lipid profile, and other metabolic indices) were analyzed at the Core Laboratory of Hospital Clínic–IDIBAPS.

### 2.10. Statistical Analysis

Descriptive statistics for baseline characteristics were calculated as means and standard deviations for quantitative variables, and as frequencies and percentages for categorical variables. Temporal stability of potential confounders was examined using linear mixed models, including time as a fixed effect (four assessment time points) and participant as a random effect. Variables tested included dietary intake (total energy, carbohydrates, protein, fat, fiber, vitamin C, carotenoids, and overall fruit and vegetable consumption), moderate-to-vigorous physical activity, time in bed, and body composition (weight, BMI, body fat percentage, and muscle mass). Baseline differences between interventions and within-intervention changes (baseline to final) in plasma lycopene, BDNF concentrations, cognitive outcomes, and potential confounders were assessed using paired t-tests or Wilcoxon signed-rank tests, depending on data distribution.

To compare changes in plasma lycopene, BDNF concentrations, and cognitive outcomes between interventions, linear mixed models were fitted with intervention, period, and baseline values as fixed effects, and participant as a random effect. Interaction terms were tested to determine carry-over effects (intervention × period) and sex-specific effects (intervention × sex). When a potential carry-over effect was detected, the model was adjusted by including a carry-over term that assumed no residual effect in the first period and carry-over in participants who received the tomato intervention first. Sex-specific interactions were excluded from the final models as they were not statistically significant. When model assumptions of normality or homoscedasticity were violated, robust variance estimation was applied. To account for multiple testing across the cognitive outcomes, false discovery rate (FDR) correction was applied using the Benjamini–Hochberg procedure separately within each validated cognitive instrument (d2-R, M-WCST, and FNAME). This domain-wise approach was selected because subscores within each test assess conceptually and empirically correlated facets of the same cognitive construct, whereas the three instruments measure distinct cognitive domains. Standardized effect sizes (Cohen’s *d*) were calculated by dividing the adjusted mean difference between interventions, obtained from the mixed-effects models, by the residual SD of each model. Although the a priori sample size calculation was based on BDNF, post hoc power was computed for the cognitive co-primary endpoints using the observed standardized effect sizes and the achieved sample size (*n* = 42), with α = 0.05 (two-sided).

As the objective of the study was to assess the effect of tomato consumption in healthy adults, and inclusion criteria were verified by self-report, sensitivity analyses were conducted to address potential post-inclusion deviations: (1) excluding participants with mean triglycerides >150 mg/dL (*n* = 2); (2) excluding participants with mean BMI >30 kg/m^2^ (*n* = 1); (3) excluding participants meeting either criterion (*n* = 3); (4) including those who completed only one study period (*n* = 3); and (5) restricting analyses to the first study period, treating it as a parallel-group design and using non-paired comparisons, with models adjusted for age, sex, and education level.

A receiver operating characteristic (ROC) curve was generated to determine the optimal cut-off of plasma lycopene changes for discriminating between tomato and control interventions. Per-protocol analyses were also performed based on plasma lycopene changes: (1) excluding participants with reduced or no change (*n* = 7) in plasma lycopene after the tomato intervention; (2) excluding those with substantial increases in plasma lycopene after the control intervention (*n* = 9); and (3) excluding participants who met either criterion (*n* = 14).

For fMRI scans, independent component analysis (ICA) was used to identify resting-state brain networks, followed by dual regression to estimate subject-specific functional network distribution. Voxel-wise comparisons were conducted using Randomize, the non-parametric permutation tool in FSL [35]. Baseline-to-final changes were assessed separately for the tomato (*n* = 13) and control (*n* = 12) interventions. Statistical significance was tested using threshold-free cluster enhancement (TFCE), and family-wise error (*p* < 0.05) to correct for multiple comparisons. Results obtained with only TFCE correction were also reported at *p* < 0.005, with a minimum cluster size of 50 voxels.

For clusters showing significant effects, average connectivity values were extracted per subject and time point. Correlations between these values and changes in plasma lycopene, BDNF concentrations, and cognitive performance, were evaluated using pooled data from both intervention periods. Pearson or Spearman coefficients were applied as appropriate. Dose–response relationships between changes in plasma lycopene and changes in cognitive domains were further examined using linear mixed models with plasma lycopene change, baseline cognitive performance, and study period as fixed effects, and participant as a random effect. Statistical analyses were performed using Stata version 15.1 and RStudio version 2024.12.1 (Build 563), employing the R packages Hmisc, dplyr, tidyr, ggplot2, and ggtext for data processing, correlation analyses, and visualization. All tests were two-sided with significance set at *p* < 0.05.

## 3. Results

### 3.1. Carotenoid Profile of Tomato Paste

Total carotenoid concentration in the tomato paste was 259.2 ± 9.4 mg/100 g fresh weight (FW; mean ± SE). Lycopene was the most abundant carotenoid (111.1 ± 4.5 mg/100 g FW), followed by phytoene (96.7 ± 8.2 mg/100 g FW), β-carotene (45.0 mg/100 g FW), and α-carotene (6.5 ± 0.9 mg/100 g FW). Lycopene was predominantly present as all-*E* isomers (83.1 ± 4.3 mg/100 g FW), at nearly three times the concentration of *Z* isomers (28.0 ± 1.4 mg/100 g FW). Further details on the physicochemical composition and nutritional profile of the tomato paste are presented in Table 1.

### 3.2. Baseline Characteristics

**Figure 1:**
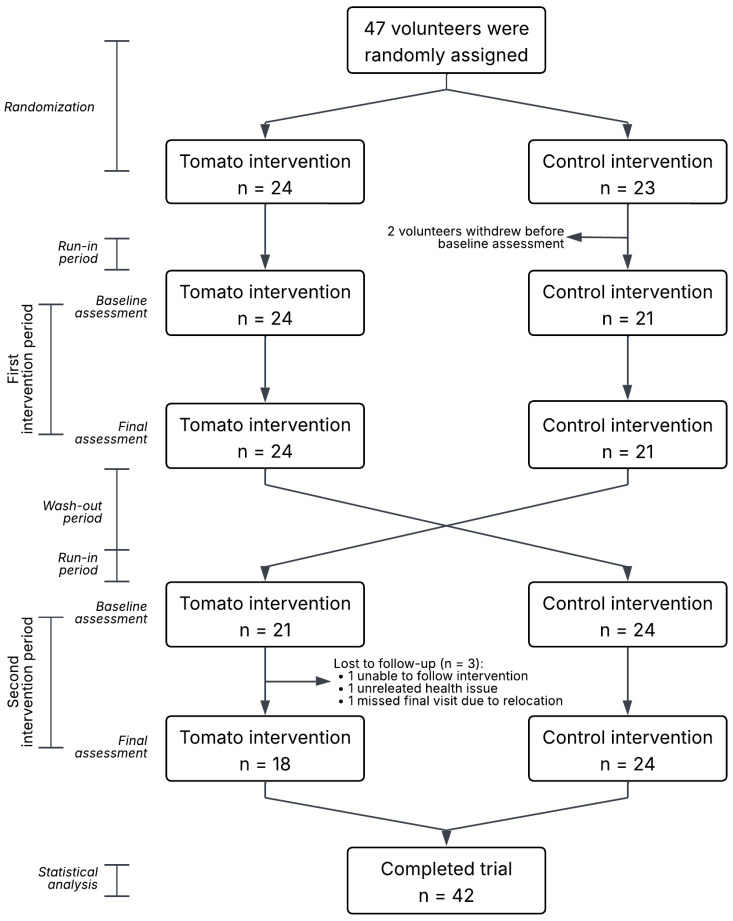
Flowchart of participant progression through the randomized crossover trial.

Five of 47 participants (10.6%) discontinued the study. Participants who discontinued were descriptively comparable to those who completed the study in baseline characteristics (Supplementary Data, Table S1). Withdrawals occurred before study initiation (*n* = 2) due to non-study-related health issues (*n* = 1), relocation (*n* = 1), or difficulties adhering to the protocol (*n* = 1) (Figure 1). Baseline characteristics of the 42 participants who completed the study are presented in Table 2. Participants were 46.6 ± 4.8 years old (mean ± SD); 66.7% were women; 64.3% were married; and 92.9% had completed higher or postgraduate education. They engaged in 305.8 ± 50.8 min/day of moderate-to-vigorous physical activity, had an energy intake of 2347.4 ± 584.8 kcal/day, and a BMI of 25.1 ± 3.4 kg/m^2^. Baseline biochemical and clinical parameters were within normal ranges: fasting glucose 86.3 ± 6.9 mg/dL, total cholesterol 193.7 ± 30.1 mg/dL, triglycerides 85.1 ± 35.1 mg/dL, and systolic/diastolic blood pressure 114.8 ± 13.9 and 77.3 ± 9.9 mmHg, respectively.

No significant changes were observed in lifestyle variables, including dietary intake, moderate-to-vigorous physical activity, time in bed, and body composition (all *p* > 0.05; Supplementary Data, Table S2), suggesting that no relevant concomitant lifestyle changes occurred during the intervention periods. No adverse events related to the intervention were reported during the trial.

### 3.3. Tomato Intake, Plasma Lycopene Response, and Adherence

During the tomato intervention, the average intake of concentrated tomato paste was 34.6 ± 6.4 g, providing 38.4 ± 7.1 mg/day of lycopene.

Plasma lycopene concentrations are presented in Table 3. Although baseline levels differed significantly between interventions, no significant carry-over effects were detected statistically. Lycopene concentrations remained stable during the control period but increased markedly with tomato intake, nearby doubling from baseline (mean change 0.40 µmol/L; 95% CI 0.30, 0.49). Between-intervention comparisons confirmed this effect, with an estimated mean difference of 0.34 µmol/L (95% CI 0.24, 0.43), corresponding to a large standardized effect (Cohen’s *d* = 1.60).

Changes in plasma lycopene served as an objective biomarker of adherence (area under the ROC curve = 0.85, 95% CI 0.77, 0.94; Supplementary Data, Figure S2). Using a ROC-derived cut-off of 0.14 µmol/L (83% sensitivity, 79% specificity), where values above the threshold were expected during the tomato intervention and below the threshold during the control period, we identified non-adherence in 14 participants: five during the tomato intervention, seven during the control period, and two during both.

### 3.4. Changes in Plasma Brain-Derived Neurotrophic Factor (BDNF)

Plasma BDNF concentrations are shown in Table 3. Mixed models revealed a carry-over effect for BDNF concentrations, which may account for the baseline differences between interventions, as participants who received the tomato intervention in the first period began the control period with elevated BDNF levels. After adjustment for carry-over, tomato paste intake resulted in a mean increase of 15.2 ng/mL in plasma BDNF compared with the control intervention (95% CI: 0.02, 30.4), although this finding should be interpreted cautiously given the borderline statistical significance, high inter-individual variability, and observed carry-over effect. To assess the influence of carry-over, we conducted a sensitivity analysis using first-period data only (parallel design approach). Results were consistent, showing a mean difference of 11.0 ng/mL (95% CI: 0.9, 21.2; Supplementary Data, Table S3).

### 3.5. Changes in Cognitive Performance

Table 3 presents baseline and post-intervention values for all cognitive outcomes by intervention. Baseline cognitive function test scores did not differ significantly between interventions, except for perseverative errors on the M-WCST. While no changes were observed during the control period, tomato paste intake improved selective attention and associative memory, with significant gains in concentration performance (estimated mean change 7.8 points; 95% CI: 3.0, 12.5) and processing speed (8.5 points; 95% CI: 2.2, 14.9) on the d2-R test, as well as face-name matching (0.8 points; 95% CI: 0.1, 1.5) and total score (1.3 points; 95% CI: 0.2, 2.3) on FNAME. In contrast, executive function was unaffected by either intervention.

Crossover analysis (between-intervention comparisons) confirmed significant improvements with tomato paste intake for concentration performance (mean difference 7.2 points; 95% CI: 1.4, 12.9), processing speed (8.3 points; 95% CI: 1.6, 15.0), and face-name matching (0.8 points; 95% CI: 0.2, 1.3), with standardized effects of moderate magnitude (Cohen’s *d* = 0.53–0.61) and post hoc power ≥92% for all three outcomes. These effects remained significant after domain-wise FDR correction (all *q* < 0.05; Table 3).

These findings remained robust across multiple sensitivity analyses, including the exclusion of participants who developed metabolic deviations during the study (e.g., elevated triglycerides or BMI), the inclusion of those who completed only one intervention period, and the first-period-only analysis (Supplementary Data, Tables S3 and S4). Per-protocol analyses excluding participants with inadequate adherence further amplified the effects of tomato consumption compared with control on concentration performance (13.7 points; 95% CI: 6.5, 20.9; *p* < 0.001) and processing speed (14.7 points; 95% CI: 6.5, 22.9; *p* < 0.001; Supplementary Data, Table S5).

### 3.6. Changes in Brain Connectivity

**Figure 2:**
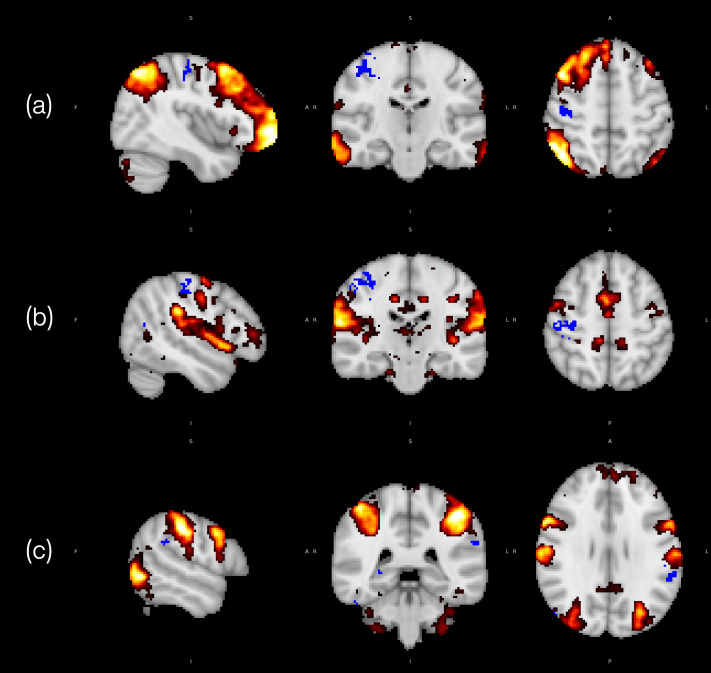
Functional connectivity patterns after tomato and control interventions (uncorrected *p* < 0.005). Clusters displaying reduced connectivity are shown in blue; red-yellow represents the functional network in which the differences were detected. Changes after tomato intervention: (**a**) Right frontoparietal network: cluster in the right precentral and postcentral gyri showing reduced connectivity. (**b**) Auditory network: cluster extending over the right postcentral gyrus, precentral gyrus, and supramarginal gyrus, showing reduced connectivity. Changes after control intervention: (**c**) Dorsal attention network: cluster involving the left supramarginal gyrus and the parietal operculum cortex showing reduced connectivity.

Baseline characteristics of the neuroimaging subsample (*n* = 14) are presented in Supplementary Data (Table S6) and were overall similar to those of the remaining study participants. Voxel-wise analyses (uncorrected *p* < 0.005) revealed distinct changes in large-scale brain networks identified through ICA. Tomato intake reduced connectivity within the right frontoparietal and auditory networks, specifically with sensorimotor and supramarginal regions (Figure 2a,b). In contrast, the control intervention weakened connectivity within the dorsal attention network, particularly in the supramarginal and parietal operculum cortices (Figure 2c).

### 3.7. Lycopene, BDNF, Cognition and Connectivity

**Figure 3:**
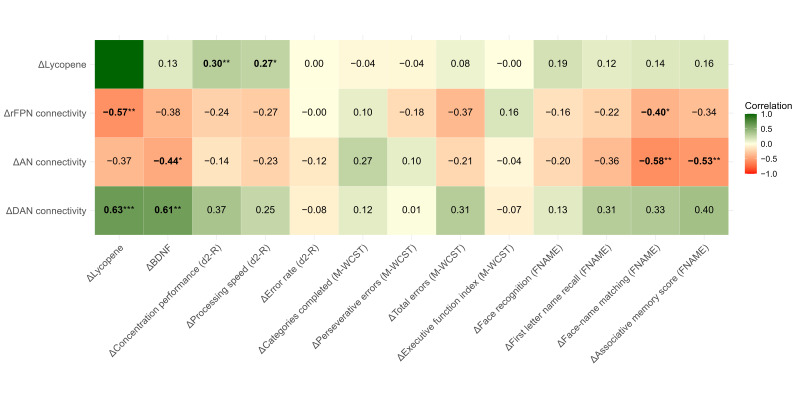
Correlations between changes in plasma lycopene, BDNF concentrations, cognitive performance, and resting-state functional brain connectivity. Pearson or Spearman correlation coefficients were calculated depending on variable distribution. Δ values represent within-subject changes from baseline to final assessment in each intervention period; data from both conditions were pooled, resulting in two observations per participant. *p* < 0.05 (*), *p* < 0.01 (**), *p* < 0.001 (***). AN, auditory network; BDNF, brain-derived neurotrophic factor; DAN, dorsal attention network; FNAME, Face-Name Associative Memory Exam; M-WCST, Modified Wisconsin Card Sorting Test; rFPN, right frontoparietal network.

Correlation analyses are shown in Figure 3. Increased plasma lycopene was modestly correlated with improvements in concentration performance (r = 0.30, *p* = 0.006) and processing speed (r = 0.27, *p* = 0.012), associations further supported by dose–response analyses (Supplementary Data, Figures S3 and S4), showing that each 1 µmol/L increase in plasma lycopene was associated with improvements of 9.7 points in concentration performance (95% CI: 0.7, 18.6; *p* = 0.034) and 12.4 points in processing speed (95% CI: 1.4, 23.4; *p* = 0.028), with statistically significant improvements starting from increases of 0.13 µmol/L and 0.19 µmol/L, respectively.

In addition, increased lycopene levels were moderately correlated with enhanced connectivity in the dorsal attention network (r = 0.63, *p* < 0.001), reduced connectivity in the right frontoparietal network (r = −0.57, *p* = 0.003), and showed a non-significant trend toward lower connectivity in the auditory network (r = −0.37, *p* = 0.069).

Changes in plasma BDNF were not associated with cognitive outcomes but showed moderate associations with functional brain connectivity. BDNF increases were negatively associated with the auditory network connectivity (ρ = −0.44, *p* = 0.026), and positively associated with the dorsal attention network (ρ = 0.61, *p* = 0.001), with a non-significant negative trend with the right frontoparietal network (ρ = −0.38, *p* = 0.063).

Connectivity changes were also correlated with cognitive gains, with associations of modest to moderate magnitude. Reduced right frontoparietal and auditory connectivity were correlated with better performance in the associative memory task (face–name matching; ρ = −0.40, *p* = 0.048; and ρ = −0.58, *p* = 0.002, respectively). Additionally, reduced auditory connectivity correlated with higher associative memory scores (r = −0.53, *p* = 0.007), with a similar but non-significant trend for the right frontoparietal network (r = −0.34, *p* = 0.094). No significant correlations emerged for the dorsal attention network, although positive trends were observed for concentration performance (r = 0.37, *p* = 0.070) and associative memory (r = 0.40, *p* = 0.051).

Although changes in plasma lycopene were not correlated with changes in BDNF when combining both periods, moderate associations were observed when analyzing the first study period only (ρ = 0.42, *p* = 0.006) and at baseline (ρ = 0.47, *p* = 0.002).

## 4. Discussion

This randomized crossover trial demonstrates that daily intake of tomato paste (~35 g; ~38 mg of lycopene) enhances selective attention and associative memory in healthy middle-aged adults. These cognitive improvements, accompanied by increases in plasma lycopene and a borderline increase in BDNF, were further supported by resting-state connectivity changes across frontoparietal, auditory, and dorsal attention networks, suggesting that tomato bioactive compounds may promote neurotrophic effects and network-specific modulation of brain function.

### 4.1. Previous Evidence on Tomato and Cognition

Our findings align with observational research reporting better cognitive function among individuals with higher tomato consumption [11] and higher lycopene intake, as assessed through dietary questionnaires or plasma concentrations [12,13,36,37]. Lower circulating carotenoid concentrations have also been observed in patients with dementia compared with cognitively healthy controls [38,39], as well as in individuals showing an accelerated rather than delayed brain-aging phenotype [5].

However, interventional evidence on the cognitive effects of tomato intake remains limited. Only a few prior studies have included tomato or lycopene, but these were embedded with multicomponent interventions. Consistent with our results, a recent three-arm pilot study in older adults found that a carotenoid-rich functional food containing tomato powder (~3.4 μg and ~7.2 μg lycopene in the intervention arms) improved processing capacity, attention, and working memory over eight weeks compared with placebo [17], though direct comparison is limited by differences in cognitive tests. In a five-week crossover study, a mixed berry beverage containing tomato powder improved working memory in adults aged 50–70 years but not selective attention, a contrast with our findings. This discrepancy may reflect the practice effects reported in that study from repeated exposure to the same cognitive test [14].

Similarly, in a 16-week crossover trial, Carrillo et al. [18] reported that a mixed fruit and vegetable nutraceutical, including tomato and lycopene, enhanced attention, executive function, and global cognition, accompanied by increases in BDNF. Crosta et al. [16] likewise showed that an eight-week intervention combining bacopa, lycopene, astaxanthin, and vitamin B12 improved executive function in older adults. Although we did not observe changes in executive function using the M-WCST, the Trail Making Test (TMT) used in those studies may be more sensitive to concentration and processing-speed demands [40], domains in which we observed improvements in the d2-R. Importantly, the multicomponent nature of previous interventions precludes attribution of the effects specifically to tomato or lycopene.

In contrast, a four-week crossover trial of watermelon juice (14.4 mg/day lycopene) in postmenopausal women (mean age 60 years) found no cognitive effects [15], potentially due to its shorter duration, lower lycopene dose, small sample size (*n* = 16), and brief washout period (two weeks), which resulted in a carry-over effect in lycopene concentrations.

### 4.2. Brain Network Connectivity and Cognition

In the present trial, changes in resting-state connectivity provide mechanistic context for the observed cognitive benefits. The reduced connectivity within the right frontoparietal network following the tomato intervention is consistent with prior reports linking lower connectivity in this network to faster response times in high-load working memory tasks [41]. Although our study did not directly assess working memory, this cognitive domain partially overlaps with selective attention [42,43], where we observed improvements. The frontoparietal network plays a central role in top-down attentional control, modulating sensory regions to prioritize goal-relevant information [43,44], a mechanism likely engaged during performance on the d2-R attention tasks. Given the strong influence of attention on memory encoding [43], this mechanism may also help explain the parallel improvements in associative memory observed in our study.

Similarly, the reduction in auditory network connectivity may represent a complementary mechanism supporting cognitive optimization. Auditory attention relies on top-down processes that enhance relevant signals and suppress distractors, thereby facilitating task performance [43]. Reduced connectivity within this network may therefore reflect reduced auditory interference, improving processing efficiency during visually demanding tasks. Together, these neural changes suggest that tomato intake promotes functional reorganization of large-scale brain networks, enhancing neural efficiency and adaptability through the pruning of redundant cross-network interactions. Similar adaptations, including decreased frontoparietal connectivity, have been observed following working memory training interventions [45].

Conversely, reduced connectivity within the dorsal attention network following the low-lycopene control intervention may also help explain between-intervention differences in selective attention and associative memory, given this network’s essential role in sustaining attentional control [46].

### 4.3. Associations Between Lycopene, BDNF, Cognition, and Brain Connectivity

Our findings support observational associations previously reported between lycopene levels and cognitive performance [12,13,36]. Increases in plasma lycopene correlated with improvements in concentration and processing speed, consistent with results from the EVA study, which showed that lower lycopene levels were associated with poorer performance on the Digit Symbol Substitution test, a measure of sustained attention and processing speed [13]. In contrast to observational studies, we did not observe direct correlations between lycopene concentrations and executive function [13] or memory [12,36]. These discrepancies may reflect methodological differences across studies, and the use of distinct cognitive assessments that limit direct comparability.

To our knowledge, this is the first clinical trial to link changes in the antioxidant lycopene with alterations in functional brain connectivity. Specifically, increased lycopene correlated with reduced connectivity in the right frontoparietal network and enhanced connectivity in the dorsal attention network, the latter consistent with prior observational evidence that lycopene modulates this network [36]. The parallel associations between plasma lycopene, BDNF and network connectivity suggest a shared neurotrophic mechanism underlying functional brain reorganization following tomato consumption, in line with previous findings linking BDNF to connectivity changes [47]. However, in this crossover design, changes in lycopene concentrations were not correlated with changes in BDNF, possibly reflecting variability or carry-over effects. Notably, baseline associations and analyses restricted to the first study period suggest a potential relationship between lycopene status and circulating BDNF under carry-over-free conditions. Furthermore, the observed correlations between connectivity changes and cognitive gains provide additional convergent evidence suggestive of the biological coherence of our findings.

### 4.4. Neuroprotective Properties of Tomato Compounds

The neuroprotective properties of tomato bioactive compounds provide biological plausibility for our observations. In the present study, tomato intake may have increased circulating BDNF, thereby supporting a neurotrophic mechanism consistent with preclinical evidence showing that lycopene upregulates BDNF expression and enhances synaptic plasticity [9,10,48]. This effect may be partly explained by the antioxidant properties of tomato-derived compounds, since oxidative stress and neuroinflammation are known to suppress BDNF expression, and their attenuation by lycopene may promote a cellular environment conducive to neurotrophic signaling [8,19]. At the molecular level, lycopene-mediated neuroprotection has been linked to the inhibition of inflammatory signaling pathways, including NF-κB, JNK, iNOS, COX-2, and NOX2, together with activation of Nrf2-related antioxidant responses and anti-apoptotic mechanisms [49]. Notably, despite the one-month washout, a residual carry-over effect was detected in BDNF concentrations, potentially reflecting sustained neurotrophic effects following tomato intake, given that BDNF regulation involves long-term neuronal and molecular adaptations [10,48].

Beyond lycopene, tomatoes contain a wide array of bioactive compounds, including other carotenoids (e.g., phytoene, β-carotene, and lutein), vitamins (A, C, and E), and (poly)phenols (e.g., naringenin, quercetin, caffeic acid, kaempferol, chlorogenic acid, rutin) [6], that may jointly contribute to cognitive improvements through their antioxidant and anti-inflammatory activities [18]. In addition, tomato consumption may indirectly influence brain health through the microbiota–gut–brain axis by promoting beneficial gut microbiota profiles and the production of neuroactive metabolites [17,50,51,52].

### 4.5. Strengths and Limitations

By targeting healthy middle-aged adults rather than older populations, where cognitive improvements are more easily detected, this study enables earlier dietary intervention and frames tomato intake as a preventive strategy for cognitive health. Unlike observational studies that rely on dietary recalls or biomarkers of intake, our controlled design provides interventional evidence and integrates cognitive testing with neuroimaging to better elucidate dietary effects on brain function. Our single-food approach allows direct attribution of the observed effects to tomato consumption, since previous clinical trials have typically incorporated multiple active ingredients. The exploratory neuroimaging sub-study, consistent in scale with previous dietary fMRI studies [53], provided convergent evidence linking connectivity changes to plasma biomarkers and cognitive outcomes, reinforcing the robustness of the results. ROC curve analyses further confirmed that plasma lycopene is a highly discriminative biomarker of adherence, supporting the reliability of per-protocol analyses. The crossover design inherently reduces inter-individual variability and strengthens internal validity by allowing each participant to serve as their own control. Although the risk of attrition bias cannot be completely excluded, withdrawals were mostly unrelated to the study interventions, suggesting a low likelihood of attrition-related bias.

Nonetheless, some limitations should be acknowledged. First, recruitment through voluntary participation yielded a sample with predominantly high educational attainment, which may limit generalizability and introduce self-selection bias. Second, inclusion criteria were verified using self-reported screening, leading to the enrollment of some participants who did not fully meet criteria, mainly due to undiagnosed metabolic conditions or inaccuracies in self-reported anthropometric data. Third, due to the unblinded nature of the study, expectancy effects cannot be fully ruled out, as participants’ awareness of the intervention may have influenced motivation or engagement during cognitive testing. However, cognitive outcomes were based on standardized performance measures, and the high concentration demand of the d2-R, use of alternate FNAME versions, and adjustment for study period help to mitigate learning- and expectancy-related influences. Moreover, concordant changes in plasma lycopene and brain connectivity, together with per-protocol and dose–response analyses, support the results and reduce the likelihood of a purely expectancy-driven explanation. Fourth, because of the whole-food nature of the intervention, a placebo matching the organoleptic and nutritional characteristics of tomato paste was not feasible. An active dietary control was therefore selected to ensure a marked contrast in lycopene exposure between intervention periods. However, this design does not allow us to fully exclude the possibility that the dietary restriction itself contributed to the observed between-intervention differences. Fifth, measures of executive function assessed through the M-WCST may not have been sufficiently sensitive to detect subtle changes in this healthy cohort. Finally, the presence of carry-over effects in BDNF concentrations indicates that the crossover design may be suboptimal for neurotrophic outcomes, which may also have influenced related neurofunctional measures. Although carry-over correction and first-period sensitivity analyses supported the observed effect, the BDNF finding should be interpreted as preliminary, given its borderline statistical significance and high inter-individual variability.

### 4.6. Future Research Directions

Future tomato-based trials should expand the cognitive battery to include domains not evaluated here, particularly those linked to auditory–frontal circuitry, such as auditory selective attention, verbal working memory, and inhibitory control, given the involvement of auditory network-related regions observed in this study. Notably, earlier studies assessing tomato or lycopene interventions did not evaluate associative memory, a domain in which we found improvements using FNAME, a tool considered sensitive to subtle cognitive changes during aging [54], underscoring the need for further targeted research. Given the carry-over effect observed for BDNF, future studies should consider longer washout periods adapted to the slower kinetics of neurotrophic biomarkers to confirm the effects of tomato intake on BDNF regulation. Task-based fMRI, targeted and untargeted metabolomic approaches, together with microbiota composition, would further help clarify the neural dynamics and biological pathways underlying tomato’s effects on cognition. Replication in larger and longer-term studies, as well as across different population subgroups, is warranted before broader generalization of these findings and to determine whether the observed effects are sustained over time and translate into clinically meaningful protection against cognitive aging.

## 5. Conclusions

Regular tomato consumption appears to enhance selective attention and associative memory in healthy middle-aged adults, potentially through antioxidant-mediated neurotrophic and neuroprotective mechanisms and targeted reorganization of functional brain networks. The specificity of these effects illustrates the capacity of nutritional interventions to modulate distinct cognitive processes and circuits. Together, these findings suggest that tomato consumption may help promote cognitive function in midlife.

## Figures and Tables

**Table 1:** Physicochemical and nutritional composition of the tomato paste.

	Values Per 100 g of Product
**Physicochemical characteristics ^1^**	
Soluble solids, °Brix	28–30
Color, a/b index	≥1.95 (intense red)
pH	4.0–4.5
Acidity, g of citric acid	1.1–2.7
Ash content, g	2.7
Moisture, g	72.5
**Nutritional information ^1^**	
Energy, kcal	92
Fat, g	0.5
Saturated fat, g	0.2
Monounsaturated fat, g	<0.1
Polyunsaturated fat, g	0.3
Carbohydrates, g	14.9
Sugars, g	14.8
Fiber, g	5.0
Protein, g	4.4
Sodium, g	0.1
**Carotenoids ^2^**	
Total carotenoid, mg; mean (SE)	259.2 (9.4)
Total lycopene, mg; mean (SE)	111.1 (4.5)
All-*E*-lycopene, mg; mean (SE)	83.1 (4.3)
All-*Z*-lycopene, mg; mean (SE)	28.0 (1.4)
5-*Z*-lycopene, mg; mean (SE)	10.7 (0.8)
7-*Z*-lycopene, mg; mean (SE)	2.9 (0.2)
9-*Z*-lycopene, mg; mean (SE)	5.8 (0.5)
13-*Z*-lycopene, mg; mean (SE)	4.2 (0.8)
15-*Z*-lycopene, mg; mean (SE)	4.5 (0.6)
Total β-carotene, mg ^3^	45.0
β-carotene, mg ^3^	41.5
9-*Z*-β-carotene, mg; mean (SE)	3.5 (0.5)
α-carotene, mg; mean (SE)	6.5 (0.9)
Phytoene, mg; mean (SE)	96.7 (8.2)

SE, standard error. ^1^ Values provided by the manufacturer. °Brix indicates the soluble solids content (1 °Brix ≈ 1 g sucrose/100 g solution), measured by refractometry. Color was measured using the Hunter Lab color scale at 12.5 °Brix, where the a/b index represents the ratio of redness (a) to yellowness (b); higher values indicate a more intense red color. Acidity was determined by titration. pH was measured at 20 °C. ^2^ Quantified by UPLC-DAD. Carotenoid samples were analyzed in triplicate and are reported as mean ± SE, *n* = 3. ^3^ No SE is shown due to single replicate of β-carotene.

**Table 2:** Baseline characteristics.

Characteristics	*n* = 42	
**Sex; ** ***n*** ** (%)**		
Men	14	(33.3)
Women	28	(66.7)
**Age, years; mean (SD)**	46.6	(4.8)
**Marital status; ** ***n*** ** (%)**		
Single	10	(23.8)
Married	27	(64.3)
Divorced	5	(11.9)
**Educational level; ** ***n*** ** (%)**		
Secondary	3	(7.1)
Higher & Postgraduate	39	(92.9)
**Anthropometry and body composition**		
Weight, kg; mean (SD)	69.3	(12.9)
Height, cm; mean (SD)	166.3	(11.1)
BMI, kg/m^2^; mean (SD)	25.1	(3.4)
Body fat, %; mean (SD)	32.1	(9.3)
Muscle mass, %; mean (SD)	29.2	(5.1)
**Biochemical parameters**		
Glucose, mg/dL; mean (SD)	86.3	(6.9)
Total cholesterol, mg/dL; mean (SD)	193.7	(30.1)
LDL-C, mg/dL; mean (SD)	117.9	(29.1)
HDL-C, mg/dL; mean (SD)	60.1	(14.5)
Triglycerides, mg/dL; mean (SD)	85.1	(35.1)
**Clinical parameters**		
Systolic blood pressure, mmHg; mean (SD)	114.8	(13.9)
Diastolic blood pressure, mmHg; mean (SD)	77.3	(9.9)
**Physical activity and sleep**		
MVPA, min/day; mean (SD)	305.8	(50.8)
Time in bed, min/day; mean (SD)	381.6	(65.1)
**Dietary intake**		
Energy, kcal/day; mean (SD)	2347.4	(584.8)
Carbohydrates, g/day; mean (SD)	199.1	(54.7)
Protein, g/day; mean (SD)	103.8	(27.4)
Fat, g/day; mean (SD)	121.2	(37.5)
Fiber, g/day; mean (SD)	33.5	(10.1)
Vitamin C, mg/day; mean (SD)	181.5	(82.9)
Carotenoids, mg/day; mean (SD)	5.3	(2.2)
Fruit consumption, g/day; mean (SD)	225.4	(137.9)
Vegetable consumption, g/day; mean (SD)	217.4	(107.2)

BMI, body mass index; HDL-C, high-density lipoprotein cholesterol; LDL-C, low-density lipoprotein cholesterol; MVPA, moderate-to-vigorous physical activity; SD, standard deviation.

**Table 3:** Changes in plasma lycopene, BDNF concentrations, and cognitive performance in response to interventions (*n* = 42).

	Low Lycopene Diet Intervention		Tomato Intervention		Difference Between Interventions ^1^			
	**Baseline,** **Mean (SD)**	**Change,** **Mean (95% CI)**	**Baseline,** **Mean (SD)**	**Change,** **Mean (95% CI)**	**Mean (95% CI)**	***p*** ** -Value**	***q*** ** -Value ^5^**	**Cohen’s ** ***d***
**Biomarkers**								
Plasma lycopene (µmol/L)	0.40 (0.23)	0.00 (−0.07, 0.08)	0.32 (0.18) ^2^	0.40 (0.30, 0.49) ^3^	0.34 (0.24, 0.43)	<0.001	-	1.60
BDNF (ng/mL)	32.2 (20.2)	−3.3 (−10.9, 4.3)	26.5 (21.5) ^2^	2.1 (−8.2, 12.5)	15.2 (0.0, 30.4) ^4^	0.050	-	0.61
**Selective attention (d2-R test)**								
Concentration performance	171.7 (29.4)	−0.3 (−4.7, 4.0)	171.0 (36.2)	7.8 (3.0, 12.5) ^3^	7.2 (1.4, 12.9)	0.015	0.022	0.53
Processing speed	189.3 (33.7)	−0.5 (−5.3, 4.4)	188.3 (41.1)	8.5 (2.2, 14.9) ^3^	8.3 (1.6, 15.0)	0.015	0.022	0.57
Error rate	9.0 (7.9)	−0.3 (−1.6, 1.0)	8.7 (8.4)	0.0 (−1.6, 1.7)	0.5 (−1.6, 2.6)	0.650	0.650	0.11
**Executive function (Modified-Wisconsin Card Sorting Test)**								
Categories completed	5.8 (0.4)	0.0 (−0.2, 0.2)	5.9 (0.4)	0.0 (−0.1, 0.1)	0.0 (−0.1, 0.2)	0.669	0.669	0.12
Perseverative errors	0.4 (1.1)	0.2 (−0.4, 0.7)	1.0 (1.8) ^2^	−0.4 (−0.9, 0.1)	−0.1 (−0.6, 0.3)	0.612	0.669	−0.12
Total errors	3.3 (3.6)	−0.5 (−1.8, 0.9)	3.5 (3.7)	0.1 (−1.0, 1.2)	0.6 (−0.8, 1.9)	0.405	0.669	0.20
Executive function index	108.2 (7.1)	−0.2 (−3.1, 2.7)	107.2 (7.6)	0.8 (−1.0, 2.7)	0.7 (−1.9, 3.4)	0.589	0.669	0.15
**Associative memory (Face-Name Associative Memory Exam)**								
Face recognition	11.6 (0.9)	−0.1 (−0.3, 0.2)	11.6 (0.7)	0.0 (−0.2, 0.3)	0.1 (−0.1, 0.4)	0.335	0.447	0.25
First letter name recall	4.1 (2.1)	0.2 (−0.4, 0.9)	4.0 (2.0)	0.4 (−0.2, 1.1)	−0.0 (−0.7, 0.6)	0.909	0.909	−0.03
Face-name matching	9.8 (1.7)	−0.2 (−0.8, 0.4)	9.4 (2.0)	0.8 (0.1, 1.5) ^3^	0.8 (0.2, 1.3)	0.007	0.027	0.61
Associative memory score	25.5 (3.6)	−0.0 (−1.0, 0.9)	24.9 (3.5)	1.3 (0.2, 2.3) ^3^	1.0 (−0.1, 2.1)	0.084	0.168	0.41

BDNF, brain-derived neurotrophic factor; CI, confidence interval; SD, standard deviation. Post-intervention values are shown as change, indicating the direction and magnitude of within-individual changes. ^1^ Estimated mean differences between interventions (Tomato vs. Control) were obtained from linear mixed-effects models including intervention, period, and baseline values as fixed effects, and participant as a random effect. Standardized effect sizes (Cohen’s *d*) were derived from mixed-effects models as the adjusted mean difference divided by the residual SD. ^2^ Significant between-group differences at baseline (*p* < 0.05) by Wilcoxon signed-rank test. ^3^ Significantly different from baseline (*p* < 0.05) by paired t-test or Wilcoxon signed-rank test. ^4^ Including a carry-over term in the model. ^5^ *q*-values are false discovery rate-adjusted within each cognitive instrument (d2-R, M-WCST, FNAME) following the Benjamini–Hochberg procedure.

## Data Availability

Due to ethical and confidentiality constraints, the human participant data cannot be publicly shared. Limited, anonymized datasets may be requested from the corresponding author, subject to ethical approval.

## Supplementary Materials

The following supporting information can be downloaded at: https://www.mdpi.com/article/10.3390/antiox15050644/s1, Figure S1: Study design; Figure S2: Receiver operating characteristic (ROC) curve of plasma lycopene changes (Δ = final − baseline) as a biomarker of adherence; Figure S3: Dose–response relationship between changes in plasma lycopene and changes in concentration performance; Figure S4: Dose–response relationship between changes in plasma lycopene and changes in processing speed; Table S1: Attrition analysis; Table S2: Changes in dietary intake, physical activity, and body composition across the four evaluation time points; Table S3: Sensitivity analyses using data from the first period only; Table S4: Sensitivity analyses addressing post-inclusion deviations; Table S5: Per-protocol analysis based on plasma lycopene concentrations; Table S6: Baseline characteristics of the fMRI subsample compared with the remaining cohort.

## Abbreviations

AN	Auditory network
ANTs	Advanced normalization tools
AUDIT	Alcohol Use Disorders Identification Test
BDNF	Brain-derived neurotrophic factor
BHT	Butylated hydroxytoluene
BMI	Body mass index
CI	Confidence interval
COX-2	Cyclooxygenase-2
DAD	Diode array detector
DAN	Dorsal attention network
DVARS	Derivative of root mean square variance over voxels
EDTA	Ethylenediaminetetraacetic acid
ELISA	Enzyme-linked immunosorbent assay
EPI	Echo-planar imaging
FD	Framewise displacement
FDR	False discovery rate
FFQ	Food frequency questionnaire
fMRI	Functional magnetic resonance imaging
FNAME	Face-Name Associative Memory Exam
FSL	FMRIB Software Library
FW	Fresh weight
HDL-C	High-density lipoprotein cholesterol
HPLC	High-performance liquid chromatography
ICA	Independent component analysis
iNOS	Inducible nitric oxide synthase
JNK	c-Jun N-terminal kinase
LC-MS/MS	Liquid chromatography–tandem mass spectrometry
LDL-C	Low-density lipoprotein cholesterol
MNI	Montreal Neurological Institute
MoCA	Montreal Cognitive Assessment
MTBE	Methyl tert-butyl ether
MVPA	Moderate-to-vigorous physical activity
M-WCST	Modified Wisconsin Card Sorting Test
NF-κB	Nuclear factor kappa-light-chain-enhancer of activated B cells
NOX2	NADPH oxidase 2
Nrf2	Nuclear factor erythroid 2-related type 2
PTFE	Polytetrafluoroethylene
rFPN	Right frontoparietal network
ROC	Receiver operating characteristic
SD	Standard deviation
SE	Standard error
SPM	Statistical parametric mapping
TE	Echo time
TFCE	Threshold-free cluster enhancement
TMT	Trail Making Test
TR	Repetition time
UPLC	Ultra-performance liquid chromatography
