# Supplementary material for: Tomato Intake Improves Cognitive Performance and Modulates Functional Brain Networks in Healthy Adults: A Randomized Crossover Clinical Trial
Source: Antioxidants (Basel). 2026 May 19;15(5):644. doi: 10.3390/antiox15050644 (PMC13203555; doi:10.3390/antiox15050644)
Supplement: Supplementary file 1 [file antioxidants-15-00644-s001.zip › antioxidants-4289729-supplementary.pdf]

Supplementary Data

Table of contents

Figure S1. Study design 2

Figure S2. Receiver operating characteristic (ROC) curve of plasma lycopene changes (Δ = final - baseline) as a biomarker of adherence. 3

Figure S3. Dose–response relationship between changes in plasma lycopene and changes in concentration performance. 4

Figure S4. Dose–response relationship between changes in plasma lycopene and changes in processing speed. 5

Table S1. Attrition analysis 6

Table S2. Changes in dietary intake, physical activity, and body composition across the four evaluation time points 7

Table S3. Sensitivity analyses using data from the first period only 8

Table S4. Sensitivity analyses addressing post-inclusion deviations 9

Table S5. Per-protocol analysis based on plasma lycopene concentrations 10

Table S6. Baseline characteristics of the fMRI subsample compared with the remaining cohort 11

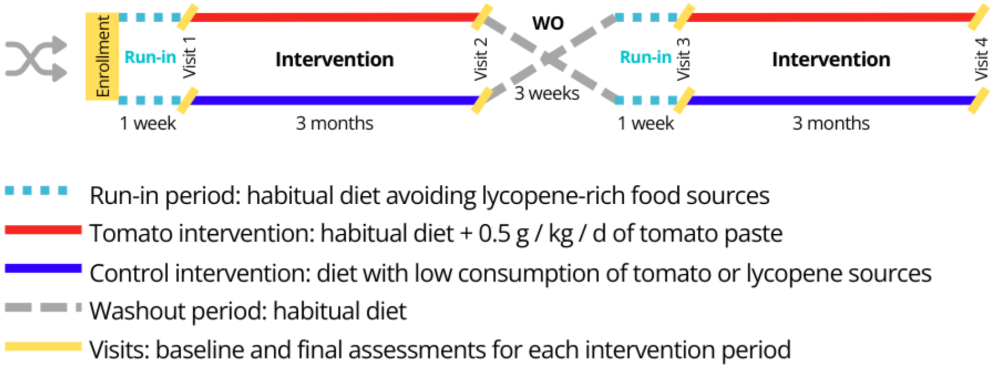

Figure S1. Study design

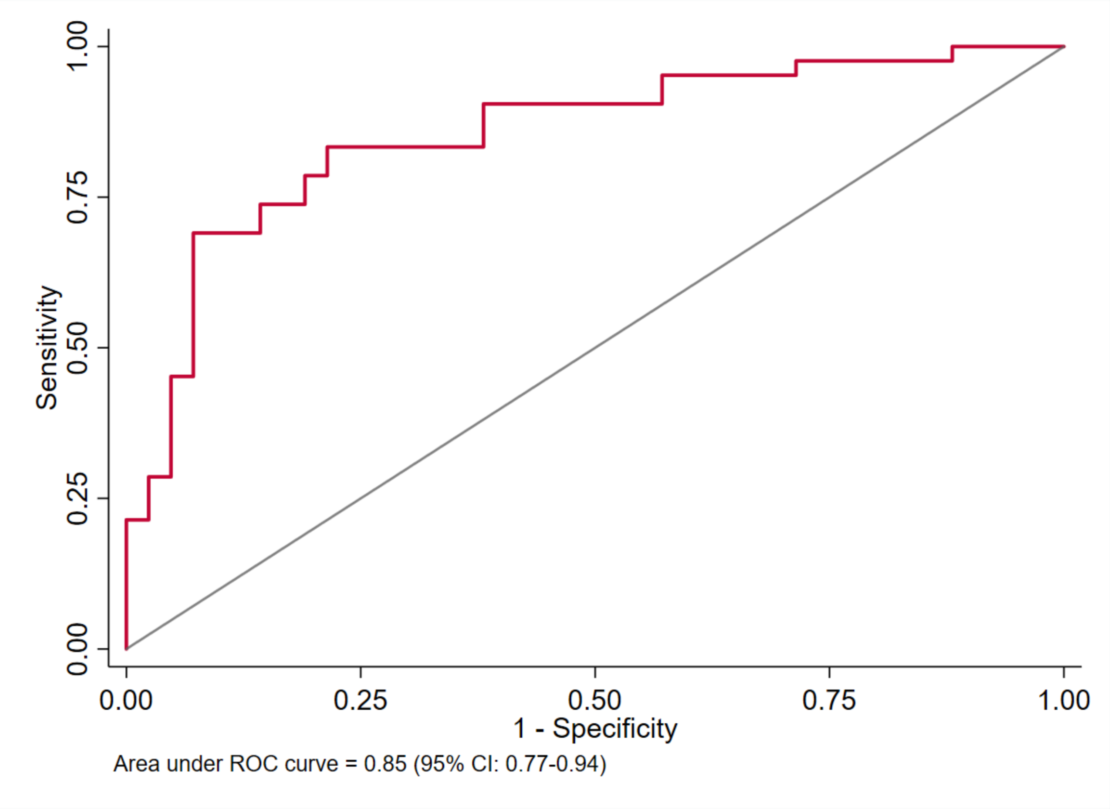

Figure S2. Receiver operating characteristic (ROC) curve of plasma lycopene changes (Δ = final - baseline) as a biomarker of adherence. The curve shows the ability of Δ-lycopene (μmol/L) to discriminate between tomato and control interventions.

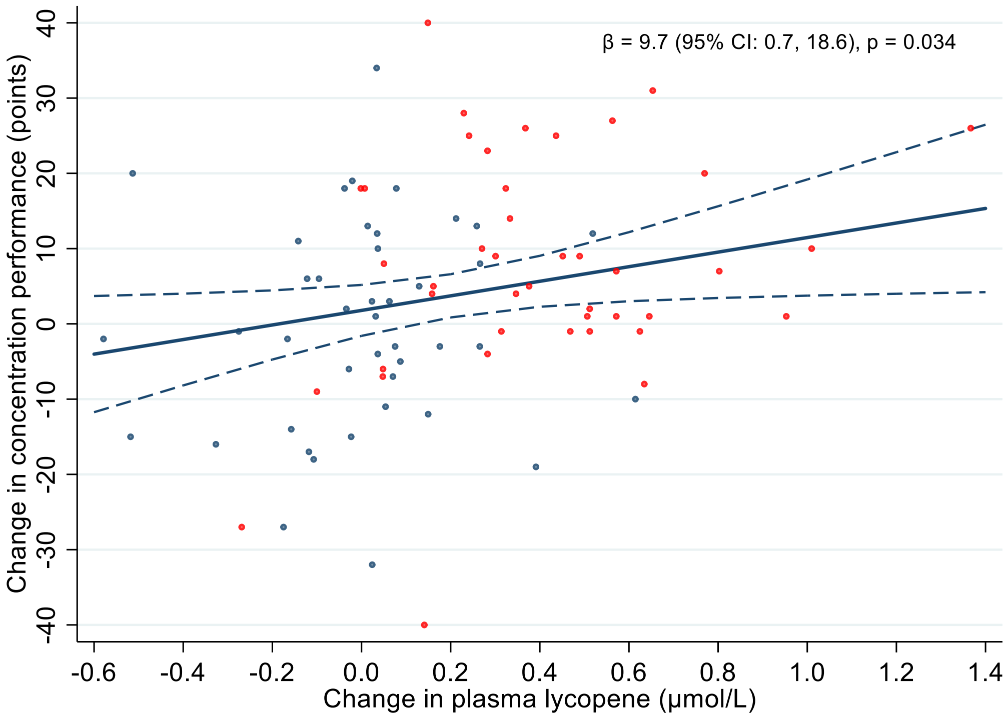

Figure S3. Dose–response relationship between changes in plasma lycopene and changes in concentration performance. Estimates were obtained using linear mixed-effects models, adjusted for baseline concentration performance and study period, with a random intercept for participants. The solid line represents the model-estimated mean, and dashed lines indicate 95% confidence intervals. Dots represent observed within-period changes, with red dots corresponding to the tomato intervention and blue dots to the control intervention. The association was positive ($\beta = 9.7$; 95% CI: 0.7, 18.6; $p = 0.034$), with statistically significant improvements in concentration performance from an increase of 0.13 μmol/L in plasma lycopene ($\beta = 3.1$; 95% CI: 0.1, 6.0; $p = 0.042$).

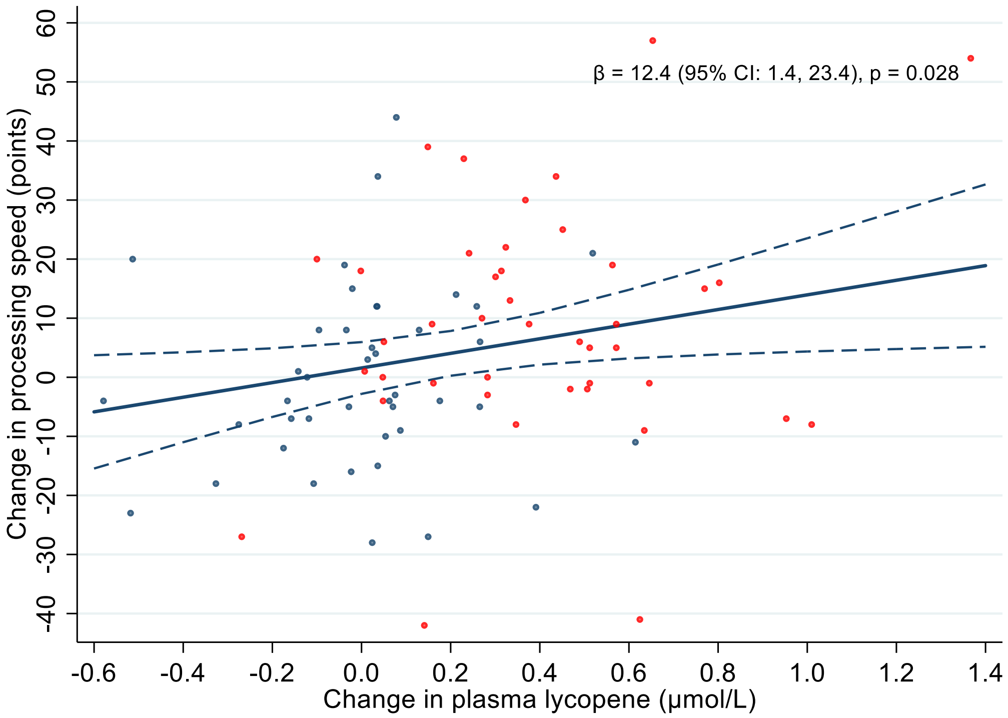

Figure S4. Dose–response relationship between changes in plasma lycopene and changes in processing speed. Estimates were obtained using linear mixed-effects models, adjusted for baseline processing speed and study period, with a random intercept for participants. The solid line represents the model-estimated mean, and dashed lines indicate 95% confidence intervals. Dots represent observed within-period changes, with red dots corresponding to the tomato intervention and blue dots to the control intervention. The association was positive (β = 12.4; 95% CI: 1.4, 23.4; p = 0.028), with statistically significant improvements in processing speed from an increase of 0.19 μmol/L in plasma lycopene (β = 3.9; 95% CI: 0.1, 7.7; p = 0.043).

Table S1. Attrition analysis

	Participants who discontinued the study (n = 5)		Participants who completed the study (n = 42)	
**Assigned sequence; n (%)**				
AB	0	(0.0)	24	(57.1)
BA	5	(100)	18	(42.9)
**Completion status; n (%)**				
Completed no period	2	(40.0)	0	(0)
Completed only period 1	3	(60)	0	(0)
Completed both periods	0	(0)	42	(100)
**Sex; n (%)**				
Men	2	(40.0)	14	(33.3)
Women	3	(60.0)	28	(66.7)
**Age, years; mean (SD)**	44.6	(4.4)	46.6	(4.8)
**Marital status; n (%)**				
Single	0	(0)	10	(23.8)
Married	5	(100)	27	(64.3)
Divorced	0	(0)	5	(11.9)
**Educational level; n (%)**				
Secondary	2	(40.0)	3	(7.1)
Higher & Postgraduate	3	(60.0)	39	(92.9)
**MoCA score; mean (SD)**	28.2	(1.3)	28.0	(1.3)
**Anthropometry; mean (SD)**				
Weight, kg	66.9	(8.5)[a]	69.3	(12.9)
Height, cm	168.6	(2.3)[a]	166.3	(11.1)
BMI, kg/m^2	23.6	(3.1)[a]	25.1	(3.4)

AB, tomato - control sequence; BA, control -tomato sequence; MoCA, Montreal Cognitive Assessment; SD, standard deviation.

[a] For two participants who discontinued the study before baseline assessment, weight and height were self-reported.

Table S2. Changes in dietary intake, physical activity, and body composition across the four evaluation time points

	Time 1<br>Baseline<br>Mean (SD)	Time 2<br>β-coefficient [a]<br>(95% CI)	Time 3<br>β-coefficient [a]<br>(95% CI)	Time 4<br>β-coefficient [a]<br>(95% CI)	*p*-value
**Dietary intake**					
Energy, kcal/day	2347.4 (584.8)	-14.0 (-113.1, 85.0)	-79.7 (-178.7, 19.3)	-72.0 (-171.0, 27.0)	0.283
Carbohydrates, g/day	199.1 (54.7)	-0.8 (-10.0, 8.4)	-5.9 (-15.1, 3.3)	-7.6 (-16.8, 1.5)	0.277
Protein, g/day	103.8 (27.4)	0.5 (-3.8, 4.9)	-1.1 (-5.5, 3.2)	-0.5 (-4.8, 3.9)	0.893
Fat, g/day	121.2 (37.5)	-0.7 (-6.6, 5.1)	-4.9 (-10.7, 0.9)	-3.9 (-9.7, 2.0)	0.284
Fiber, g/day	33.5 (10.1)	-0.3 (-1.6, 0.9)	-0.6 (-2.3, 1.1)	-1.4 (-3.2, 0.4)	0.244
Vitamin C, mg/day	181.5 (82.9)	0.2 (-15.4, 15.8)	-4.6 (-24.9, 15.8)	-16.0 (-38.2, 6.2)	0.188
Carotenoids, mg/day	5.3 (2.2)	-0.2 (-0.4, 0.1)	-0.2 (-0.7, 0.2)	-0.2 (-0.6, 0.2)	0.619
Fruit consumption, g/day	225.4 (137.9)	-7.3 (-24.6, 10.0)	-7.5 (-35.5, 20.5)	-5.1 (-40.8, 30.5)	0.868
Vegetable consumption, g/day	217.4 (107.2)	-1.7 (-14.4, 11.1)	-1.1 (-23.3, 21.0)	-11.1 (-34.7, 12.5)	0.178
**Physical activity and sleep**					
MVPA, min/day	305.8 (50.8)	-0.1 (-14.9, 14.7)	5.9 (-8.9, 20.7)	-6.5 (-21.5, 8.5)	0.447
Time in bed, min/day	381.6 (65.1)	-1.0 (-22.6, 20.5)	9.7 (-14.1, 33.6)	19.2 (-4.8, 43.2)	0.273
**Body composition**					
Weight, kg	69.3 (12.9)	0.3 (-0.2, 0.8)	0.1 (-0.4, 0.6)	0.4 (-0.1, 0.9)	0.523
BMI, kg/m²	25.1 (3.4)	-0.0 (-0.3, 0.2)	-0.1 (-0.3, 0.2)	0.0 (-0.2, 0.3)	0.853
Body fat, %	32.1 (9.3)	-0.2 (-0.8, 0.4)	-0.3 (-0.9, 0.3)	0.1 (-0.5, 0.7)	0.563
Muscle mass, %	29.2 (5.1)	0.2 (-0.2, 0.6)	0.3 (-0.1, 0.7)	0.0 (-0.4, 0.4)	0.524

BMI, body mass index; CI, confidence interval; MVPA, moderate-to-vigorous physical activity; SD, standard deviation.

[a] β-coefficients are estimated from linear mixed-effects models including time as a fixed effect and subject as a random effect. Time points 1 and 3 correspond to baseline assessments of each intervention period, while time points 2 and 4 refer to post-intervention measurements.

Table S3. Sensitivity analyses using data from the first period only

	Low lycopene diet intervention (n = 18)		Tomato intervention (n = 24)		Difference between interventions [a]	
	Baseline, mean (SD)	Change, mean (95% CI)	Baseline, mean (SD)	Change, mean (95% CI)	Mean (95% CI)	*p*-value
**Plasma lycopene (μmol/L)**	0.29 (0.20)	0.07 (-0.02, 0.16)	0.31 (0.17)	0.49 (0.35, 0.62) [b]	0.43 (0.27, 0.60)	<0.001
**BDNF (ng/mL)**	29.6 (21.7)	-7.3 (-16.5, 1.9)	30.2 (21.5)	7.5 (-10.5, 25.5)	11.0 (0.9, 21.2)	0.034
**Selective attention (d2-R test)**						
Concentration performance	167.8 (37.5)	2.6 (-5.1, 10.4)	157.1 (26.8)	12.8 (7.6, 18.1) [b]	8.7 (0.8, 16.7)	0.032
Processing speed	185.0 (38.0)	0.7 (-8.0, 9.4)	176.7 (35.5)	13.3 (4.2, 22.3) [b]	10.4 (-0.9, 21.7)	0.070
Error rate	9.6 (6.8)	-1.4 (-4.2, 1.3)	10.2 (9.9)	-0.5 (-3.1, 2.1)	0.7 (-2.3, 3.7)	0.650
**Executive function (Modified-Wisconsin Card Sorting Test)**						
Categories completed	5.7 (0.6)	0.1 (-0.6, 0.3)	6.0 (0.2)	-0.1 (-0.3, 0.1)	0.2 (-0.1, 0.5)	0.295
Perseverative errors	0.7 (1.5)	-0.2 (-1.1, 1.5)	0.9 (1.8)	-0.3 (-1.0, 0.5)	-0.5 (-1.5, 0.5)	0.305
Total errors	4.7 (4.4)	-0.4 (-3.3, 2.4)	3.5 (3.3)	0.5 (-1.0, 2.1)	0.0 (-2.2, 2.2)	0.982
Executive function index	106.7 (9.5)	-1.3 (-8.1, 5.5)	108.1 (4.8)	-0.2 (-2.8, 2.3)	2.5 (-2.8, 7.8)	0.359
**Associative memory (Face-Name Associative Memory Exam)**						
Face recognition	11.6 (1.0)	-0.1 (-0.4, 0.3)	11.8 (0.4)	-0.2 (-0.5, 0.1)	-0.1 (-0.5, 0.3)	0.538
First letter name recall	4.0 (1.9)	1.2 (0.1, 2.2) [b]	3.6 (1.6)	0.9 (0.0, 1.7) [b]	0.1 (-1.1, 1.2)	0.914
Face-name matching	10.2 (1.6)	-1.2 (-2.0, -0.4) [b]	9.2 (2.2)	0.8 (-0.2, 1.8)	1.4 (0.5, 2.3)	0.003
Associative memory score	25.8 (3.3)	-0.1 (-1.5, 1.4)	24.5 (3.1)	1.5 (0.0, 3.0) [b]	1.6 (-0.3, 3.5)	0.093

BDNF, brain-derived neurotrophic factor; CI, confidence interval; SD, standard deviation.

[a] Estimated mean differences between interventions (Tomato vs. Control) were obtained from linear models adjusted for baseline values, age, sex and educational level (Tomato vs. Control).

[b] Significantly different from baseline (p <0.05) by *t*-test or Wilcoxon rank-sum test.

No significant differences were found in baseline assessments between interventions.

Table S4. Sensitivity analyses addressing post-inclusion deviations

	Including 1-period participants (n=45)		Excluding participants with mean TG >150 mg/dL (n = 40)		Excluding participants with mean BMI >30 kg/m² (n = 41)		Excluding participants with TG >150 mg/dL or BMI >30 kg/m² (n = 39)	
	Difference between interventions [a]		Difference between interventions [a]		Difference between interventions [a]		Difference between interventions [a]	
	Mean (95% CI)	*p*-value	Mean (95% CI)	*p*-value	Mean (95% CI)	*p*-value	Mean (95% CI)	*p*-value
**Plasma lycopene (μmol/L)**	[b]	[b]	0.34 (0.24, 0.44)	<0.001	0.33 (0.24, 0.43)	<0.001	0.34 (0.24, 0.44)	<0.001
**BDNF (ng/mL)**	[b]	[b]	16.7 (1.2, 32.2) [c]	0.035	14.6 (-0.6, 29.8) [c]	0.060	16.0 (0.4, 31.5) [c]	0.044
**Selective attention (d2-R test)**								
Concentration performance	7.6 (2.0, 13.2)	0.008	6.6 (0.6, 12.6)	0.032	6.5 (0.6, 12.3)	0.030	5.8 (-0.3, 12.0)	0.061
Processing speed	8.8 (2.2, 15.3)	0.009	8.2 (1.2, 15.2)	0.021	7.3 (0.6, 14.0)	0.032	7.1 (0.1, 14.1)	0.045
Error rate	0.4 (-1.6, 2.5)	0.669	0.8 (-1.4, 3.0)	0.492	0.4 (-1.8, 2.5)	0.751	0.6 (-1.7, 2.9)	0.593
**Executive function (Modified-Wisconsin Card Sorting Test)**								
Categories completed	0.0 (-0.1, 0.2)	0.798	0.1 (-0.1, 0.3)	0.228	0.0 (-0.1, 0.2)	0.634	0.1 (-0.1, 0.3)	0.204
Perseverative errors	-0.1 (-0.6, 0.3)	0.595	-0.2 (-0.7, 0.3)	0.424	-0.1 (-0.6, 0.4)	0.581	-0.2 (-0.8, 0.3)	0.394
Total errors	0.7 (-0.6, 2.0)	0.307	0.3 (-1.1, 1.7)	0.632	0.6 (-0.8, 2.0)	0.432	0.3 (-1.1, 1.8)	0.667
Executive function index	0.5 (-2.0, 3.0)	0.695	1.7 (-0.8, 4.2)	0.183	0.8 (-1.9, 3.6)	0.554	1.9 (-0.8, 4.5)	0.162
**Associative memory (Face-Name Associative Memory Exam)**								
Face recognition	0.1 (-0.1, 0.4)	0.355	0.1 (-0.2, 0.4)	0.409	0.1 (-0.1, 0.4)	0.324	0.1 (-0.2, 0.4)	0.392
First letter name recall	-0.1 (-0.7, 0.5)	0.789	-0.1 (-0.8, 0.5)	0.750	0.1 (-0.6, 0.7)	0.852	0.0 (-0.7, 0.7)	0.988
Face-name matching	0.7 (0.2, 1.3)	0.010	0.8 (0.2, 1.3)	0.009	0.7 (0.2, 1.3)	0.010	0.7 (0.2, 1.3)	0.014
Associative memory score	0.9 (-0.2, 1.9)	0.121	0.9 (-0.2, 2.1)	0.118	1.1 (0.0, 2.3)	0.056	1.1 (-0.1, 2.3)	0.079

BDNF, brain-derived neurotrophic factor; BMI, body mass index; CI, confidence interval; TG, triglycerides.

[a] Estimated mean differences between interventions (Tomato vs. Control) were obtained from linear mixed-effects models including intervention, period, and baseline values as fixed effects, and participant as a random effect.

[b] Measured only in participants who completed both intervention periods.

[c] Including a carryover term in the model.

Table S5. Per-protocol analysis based on plasma lycopene concentrations

	Excluding participants with reduced or unchanged plasma lycopene concentrations after the tomato intervention (n = 35)		Excluding participants with marked increases in plasma lycopene after the low-lycopene phase (n = 33)		Excluding participants with reduced or unchanged lycopene after the tomato intervention, or marked increases after the low-lycopene phase (n = 28)	
	Difference between interventions [a]		Difference between interventions [a]		Difference between interventions [a]	
	Mean (95% CI)	*p*-value	Mean (95% CI)	*p*-value	Mean (95% CI)	*p*-value
**Plasma lycopene (μmol/L)**	0.41 (0.31, 0.52)	<0.001	0.40 (0.29, 0.51)	<0.001	0.50 (0.39, 0.62)	<0.001
**BDNF (ng/mL)**	17.9 (1.9, 33.9) [b]	0.028	12.5 (1.6, 23.4) [b]	0.025	15.6 (4.9, 26.2) [b]	0.004
**Selective attention (d2-R test)**						
Concentration performance	9.1 (2.7, 15.5)	0.005	9.2 (2.4, 15.9)	0.008	13.7 (6.5, 20.9)	<0.001
Processing speed	9.5 (2.0, 16.9)	0.012	10.7 (3.1, 18.3)	0.006	14.7 (6.5, 22.9)	<0.001
Error rate	0.2 (-1.7, 1.2)	0.755	0.7 (-2.2, 3.6)	0.624	0.0 (-1.8, 1.8)	0.986
**Executive function (Modified-Wisconsin Card Sorting Test)**						
Categories completed	0.1 (-0.1, 0.3)	0.165	0.1 (-0.2, 0.4)	0.457	0.2 (-0.0, 0.5)	0.094
Perseverative errors	-0.2 (-0.8, 0.4)	0.465	-0.2 (-0.8, 0.5)	0.604	-0.4 (-1.3, 0.4)	0.304
Total errors	-0.1 (-1.7, 1.4)	0.859	0.3 (-1.4, 2.0)	0.715	-0.6 (-2.4, 1.2)	0.514
Executive function index	1.8 (-1.5, 5.2)	0.285	1.4 (-2.4, 5.3)	0.463	3.11 (-1.4, 7.6)	0.176
**Associative memory (Face-Name Associative Memory Exam)**						
Face recognition	0.0 (-0.2, 0.3)	0.765	0.2 (-0.1, 0.5)	0.180	0.0 (-0.2, 0.3)	0.743
First letter name recall	-0.2 (0.9, 0.5)	0.584	0.1 (-0.6, 0.8)	0.793	-0.1 (-0.8, 0.7)	0.865
Face-name matching	0.9 (0.3, 1.5)	0.005	0.8 (0.2, 1.4)	0.005	0.9 (0.2, 1.6)	0.010
Associative memory score	0.9 (-0.4, 2.3)	0.182	1.2 (-0.1, 2.4)	0.062	1.0 (-0.4, 2.4)	0.171

BDNF, brain-derived neurotrophic factor; CI, confidence interval.

[a] Estimated mean differences between interventions (Tomato vs. Control) were obtained from linear mixed-effects models including intervention, period, and baseline values as fixed effects, and participant as a random effect.

[b] Including a carryover term in the model.

Table S6. Baseline characteristics of the fMRI subsample compared with the remaining cohort

	fMRI group (n = 14)		Participants without fMRI data (n = 28)		*p*-value
**Sex; n (%)**					0.817
Men	5	(35.7)	9	(32.1)	
Women	9	(64.3)	19	(67.9)	
**Age, years; mean (SD)**	48.3	(5.0)	45.8	(4.5)	0.116
**Marital status; n (%)**					0.121
Single	1	(7.1)	9	(32.1)	
Married	10	(71.4)	17	(60.7)	
Divorced	3	(21.4)	2	(7.1)	
**Educational level; n (%)**					0.204
Secondary	0	(0)	3	(10.7)	
Higher & Postgraduate	14	(100)	25	(89.3)	
**Anthropometry and body composition**					
Weight, kg; mean (SD)	65.7	(13.3)	71.1	(12.6)	0.204
Height, cm; mean (SD)	164.7	(10.1)	167.1	(11.7)	0.528
BMI, kg/m²; mean (SD)	24.0	(2.8)	25.7	(3.6)	0.133
Body fat, %; mean (SD)	30.0	(8.0)	33.1	(9.8)	0.318
Muscle mass, %; mean (SD)	29.9	(4.7)	28.9	(5.4)	0.552
**Biochemical parameters**					
Glucose, mg/dL; mean (SD)	84.1	(5.7)	87.3	(7.4)	0.165
Total cholesterol, mg/dL; mean (SD)	193.0	(30.7)	194.0	(30.4)	0.921
LDL-C, mg/dL; mean (SD)	113.6	(34.0)	120.0	(26.7)	0.513
HDL-C, mg/dL; mean (SD)	62.4	(15.7)	58.9	(14.1)	0.478
Triglycerides, mg/dL; mean (SD)	84.9	(24.4)	85.2	(39.9)	0.983
**Clinical parameters**					
Systolic blood pressure, mmHg; mean (SD)	114.2	(12.5)	115.1	(14.7)	0.850
Diastolic blood pressure, mmHg; mean (SD)	77.1	(9.4)	77.5	(10.3)	0.901
**Physical activity**					
MVPA, min/day; mean (SD)	311.1	(42.7)	303.1	(55.2)	0.639
Time in bed, min/day; mean (SD)	370.1	(61.2)	387.5	(67.4)	0.424
**Dietary intake**					
Energy, kcal/day; mean (SD)	2124.3	(527.7)	2458.9	(588.7)	0.080
Carbohydrates, g/day; mean (SD)	190.7	(53.7)	203.3	(55.7)	0.489
Protein, g/day; mean (SD)	89.3	(25.1)	111.0	(25.9)	0.013
Fat, g/day; mean (SD)	107.7	(33.3)	128.0	(38.2)	0.100
Fiber, g/day; mean (SD)	30.6	(8.3)	35.0	(10.7)	0.186
Vitamin C, mg/day; mean (SD)	154.1	(78.7)	195.3	(82.9)	0.131
Carotenoids, mg/day; mean (SD)	5.0	(2.3)	5.4	(2.2)	0.546
Fruit consumption, g/day; mean (SD)	193.1	(126.3)	241.6	(142.7)	0.288
Vegetable consumption, g/day; mean (SD)	181.8	(94.1)	235.2	(110.5)	0.129

BMI, body mass index; fMRI, functional magnetic resonance imaging; HDL-C, high-density lipoprotein cholesterol; LDL-C, low-density lipoprotein cholesterol; MVPA, moderate-to-vigorous physical activity; SD, standard deviation. Comparisons used *t*-tests or Mann–Whitney U tests for continuous variables and chi-squared tests for categorical variables.
